# Temporal Floristic Changes (2005–2025) Along the Lower Stretch of the Tiber River (Central Italy)

**DOI:** 10.3390/plants15050716

**Published:** 2026-02-27

**Authors:** Dario Di Lernia, Vincenzo Zuccarello, Lorenzo Pinzani, Simona Ceschin

**Affiliations:** 1 Department of Science, University of Roma Tre, Viale G. Marconi, 446, 00146 Rome, Italy; lorenzo.pinzani@uniroma3.it; 2 Department of Sciences and Biological and Environmental Technology, Salento University, St. Prov. Lecce-Monteroni, Polo Ecotekne, 73100 Lecce, Italy; vincenzo.zuccarello@unisalento.it; 3 National Biodiversity Future Center (NBFC), 90133 Palermo, Italy

**Keywords:** aquatic and riparian plants, multitemporal analysis, floristic changes, alien plants, α- and temporal β-diversity, floristic and autoecological indices, river ecosystem, Tiber River

## Abstract

A multitemporal floristic study was conducted on the aquatic and riparian plant communities of the lower stretch of the Tiber River (central Italy) to identify any floristic changes in response to possible environmental pressures that have occurred locally over time. This investigation was carried out by comparing α- and temporal β-diversity, as well as biological, chorological, and ecological traits of plant assemblages present in permanent plots (*n* = 24) and sampled at two different time points (2005, 2025). Although both aquatic and riparian plant communities showed an increase in α-diversity over time (+94.1% and +56.5%, respectively), they generally exhibited different temporal patterns. The aquatic community showed a more stable floristic structure compared to the riparian one, with a persistent dominance of eutrophic and pollution-tolerant species, although local disappearance/rarefaction of some species was recorded. On the contrary, the riparian community showed greater species turnover, mainly due to an increase in generalist, ruderal and alien species, which over time have partially replaced those typically associated with riparian habitats. Ecological trait-based analyses indicated an increase over time in the percentage of thermophilous, heliophilous and nitrophilous species in both plant communities; the riparian community also showed an increase in xerophilous ones. Overall, the results indicate that aquatic and riparian communities exhibit distinct temporal dynamics within the same river system and highlight how long-term, permanent plot-based floristic monitoring is a useful tool in environmental studies.

## 1. Introduction

River ecosystems host a peculiar flora and fauna, contributing significantly to global biodiversity [1,2,3]. They play a key role in the water, nutrient, and energy cycles, connecting terrestrial environments with coastal and marine ones [3,4]. River ecosystems also provide essential ecosystem services for humans, including water supply, food and energy resources, and recreational, sporting, and tourism activities. Despite their importance, river ecosystems are among the most vulnerable and threatened habitats, mainly due to numerous anthropogenic pressures, such as water pollution, overexploitation of resources, morpho-structural alterations (dam construction, channelization, bank reinforcement) and biological pollution [5,6,7].

The plant communities, occurring in water and along riverbanks, play a fundamental role in river ecosystems both in maintaining ecosystem balances and contributing to their stability and regulating hydrological and water self-remediation processes [8]. Moreover, due to their close relation with the water chemical and physical characteristics, they can be used as bioindicators of the ecological and qualitative status of river ecosystems, thus representing a useful tool for their study [9,10,11]. Consequently, monitoring the riverine plant communities represents a key element for understanding ecological dynamics and environmental changes that may occur along a river. In particular, multitemporal analysis of riverine plant assemblages occurring in permanent plots allows the detection of temporal plant changes in biodiversity, species composition, and morphological, chorological, and ecological traits. Such changes, assuming bioindicator value, may reveal environmental transformations that have occurred over time [12,13,14]. Furthermore, the recent introduction and development of the concept of temporal β-diversity [15,16] has provided new investigative tools for analyzing the multitemporal evolution of floristic assemblages.

The Tiber River is one of the major rivers in Italy in terms of length and habitat diversity [10,17]. Over the years, several studies have investigated its plant component from different viewpoints [7,18,19,20,21,22], constituting a valuable reference framework for performing possible diachronic botanical studies on this river.

This study is based on a multitemporal floristic comparison of permanent plots to analyze changes in α- and temporal β-diversity of aquatic and riparian plant communities along the lower stretch of the Tiber River (central Italy). These comparative temporal analyses aim to assess the floristic dynamics observed as a response to local environmental and anthropogenic pressures and, therefore, to evaluate the long-term effects of environmental changes on aquatic and riparian ecosystems.

## 2. Results

### 2.1. Temporal Plant Changes in α-Diversity and Total Coverage

In the recent floristic surveys carried out in this study along the lower stretch of the Tiber River, 33 and 180 plant species were found in the aquatic and riparian sectors, respectively. In a previous study [18], 17 aquatic species and 115 riparian species were reported. For the aquatic sector, 8.3% and 52.8% of the species were exclusive to 2005 and 2025, respectively, while 38.9% were shared between the two surveys. For the riparian sector, 13% and 44.4% of the species were exclusive to 2005 and 2025, respectively, whereas 42.5% represented the group of species common to the two surveys.

**Figure 1:**
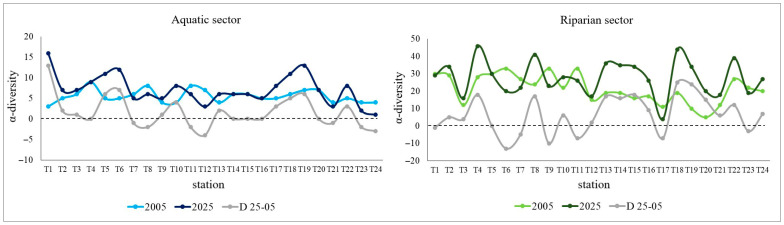
Plant α-diversity recorded in the two surveys (2005, 2025) in aquatic and riparian sectors at each station selected along the lower stretch of the Tiber River. The differential curve between the two surveys (D 25–05) is also shown, where positive and negative values indicate increase and decrease in α-diversity over time at each station, respectively. Stations are ordered along the upstream-downstream gradient of the river.

**Figure 2:**
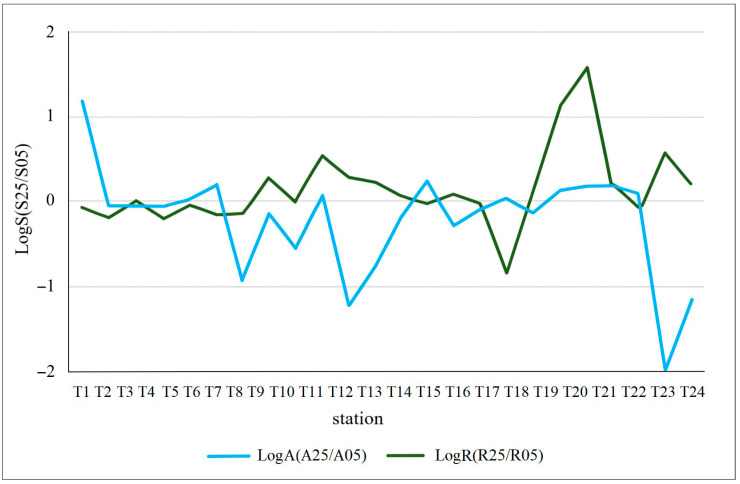
Logarithmic ratio between total plant coverage recorded at each station in 2005 and 2025 for the aquatic (LogA) and riparian (LogR) sectors. Stations are ordered along the upstream-downstream gradient of the river.

A comparison between the two floristic surveys showed that the α-diversity increased over time in both riverine sectors. This increase, moderate in the aquatic sector, is more evident in the riparian one, where a prevalence of stations showing positive values (i.e., increases) was recorded, especially in the urban stretch within Rome up to the river mouth (i.e., from T12 to T24) (Figure 1). On the other hand, when total plant coverage is considered as a parameter for comparison between the two surveys, it emerges that there has been a significant increase along the riverbanks over time (positive values prevailing over negative ones), while the opposite trend is observed in the aquatic sector (Figure 2).

### 2.2. Floristic Changes in Temporal β-Diversity Indicators

Temporal β-diversity indicators (TBI loss, TBI gain, TBI total, TBI-no change) highlight differences between the two surveys (2005, 2025) at the sampled stations, both in terms of floristic richness and relative plant coverage recorded (Table S1). Both the mean value of TBI total and TBI gain are significantly higher in the riparian sector (82.2 and 46.2, respectively) than in the aquatic one (59.0 and 21.1, respectively), whereas no significant difference was detected for TBI loss, indicating that losses in floristic richness and plant coverage are of similar magnitude in the two sectors (Table S1b).

**Figure 3:**
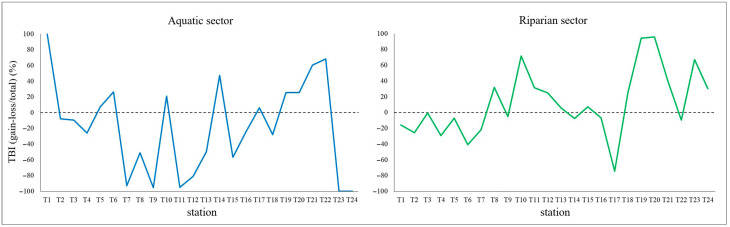
Percentage variations between TBI gain and TBI loss in aquatic and riparian sectors at each station selected along the Tiber River and ordered along the upstream-downstream gradient of the river.

By calculating the ratio between the difference in gains and losses (TBI gain-TBI loss) and total temporal β-diversity (TBI total), it was possible to assess overall percentage floristic changes over time at each station. Specifically, in the aquatic sector, the values are generally negative (Figure 3), indicating that losses (i.e., lost species and decrease in species abundance) exceed gains (i.e., new species arrived and increases in abundance of species already present in the first survey). Contrarily, in the riparian sector, positive values prevail (Figure 3), indicating that along the riverbanks there were more species arrivals than losses over time, as well as more increases than decreases in abundance of species recorded in both surveys. This pattern is particularly evident in two river sub-stretches, i.e., from T8 to T13 and from T18 to T24, where the net gain in species richness and plant coverage is more pronounced.

**Figure 4:**
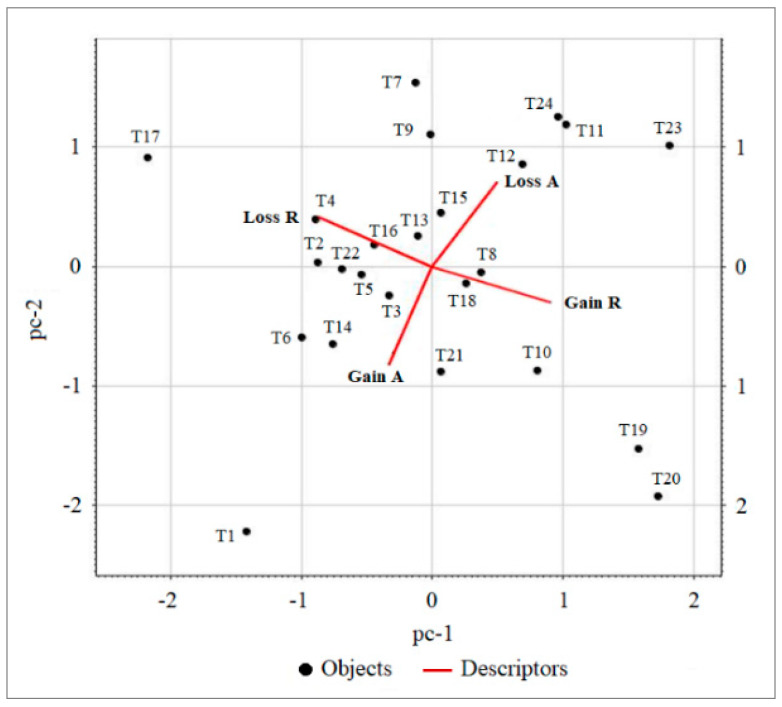
Scatterplot of PCA of the stations and variables based on TBI gain and TBI loss for the aquatic (Gain A, Loss A) and riparian (Gain R, Loss R) sectors.

The Principal Component Analysis (PCA), described by TBI gain and TBI loss values calculated for the aquatic and riparian sectors of the sampled stations (Table S1a), shows that the variables associated with the two sectors display a null correlation in the scatterplot based on the first two principal components (pc-1, pc-2), which together explain 91% of the total variance (Figure 4). TBI gain and TBI loss values within the same sector are always negatively correlated, and they are arranged almost orthogonally with respect to those of the other sector, highlighting the absence of correlation between the aquatic and riparian sectors. Consequently, the two sectors are separated and independent in the space defined by TBI indicators, despite being contiguous in the physical space of the river habitat (Figure 4).

### 2.3. Temporal Floristic Changes in Species Assemblages

The floristic data collected from the same stations sampled in 2005 and 2025 were classified based on the percentage coverage of the recorded plant species to assess how the plant species assemblages have changed over the 20-year period. Fuzzy clustering identified four main species groups for the aquatic sector and five for the riparian one. Each species group is characterized by the dominance of some species (Table 1).

**Table 1:** List of plant species with the highest coverage recorded in the two floristic surveys (2005, 2025) and characteristics of the main species groups identified in the two sectors (aquatic-a, riparian-r). Species coverage values (%) are based on the centroids of the identified groups (AAA > 25%, 25% ≤ AA ≤ 10%, 10% < A ≤ 5%).

Aquatic Sector Species	a-1	a-2	a-3	a-4	Riparian Sector Species	r-1	r-2	r-3	r-4	r-5
*Myriophyllum spicatum*	AAA		AA	A	*Phalaris arundinacea*	AA		AAA		
*Stuckenia pectinata*	AA	A	AAA		*Acer negundo*	AA		A		
*Potamogeton nodosus*	A	A	A	AAA	*Amorpha fruticosa*	AA				AA
*Ceratophyllum demersum*		AA		A	*Populus x canadensis*	AA				
*Schoenoplectus lacustris*	A				*Phragmites australis*		AAA			
					*Populus alba*		AA			AA
					*Salix alba*		A			A
					*Lythrum salicaria*				AA	
					*Echinochloa crus-galli*				AA	
					*Paspalum distichum*				A	
					*Platanus hispanica*	A		A		
					*Populus nigra*	A		A		
					*Rubus ulmifolius*		A			A
					*Alnus glutinosa*		A			A
					*Iris pseudacorus*		A			

**Figure 5:**
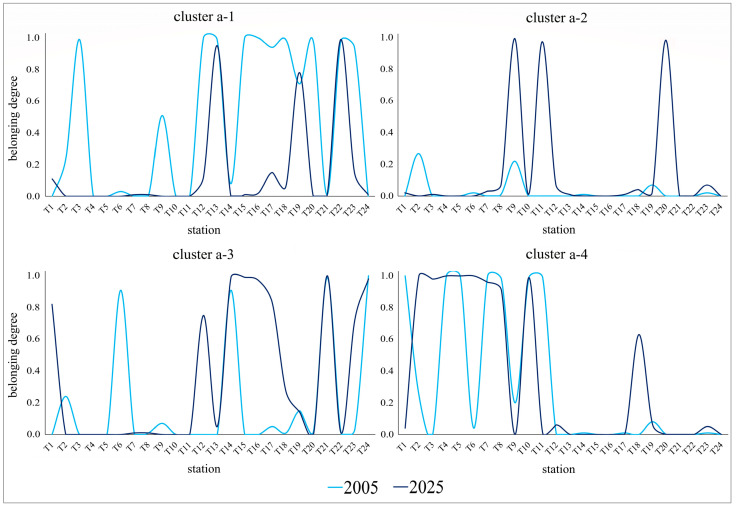
Belonging degree of each river station to the four species groups identified (fuzzy clusters) in the aquatic sector with respect to the 2005 (light blue line) and 2025 (blue line) surveys. Dominant species in the groups are: *Myriophyllum spicatum*, *Stuckenia pectinata* (a-1), *Ceratophyllum demersum* (a-2), *Stuckenia pectinata*, *Myriophyllum spicatum* (a-3), *Potamogeton nodosus* (a-4). For graphical representation, a smoothed line graph was used instead of a bar graph, for the sole purpose of facilitating data visualization.

**Figure 6:**
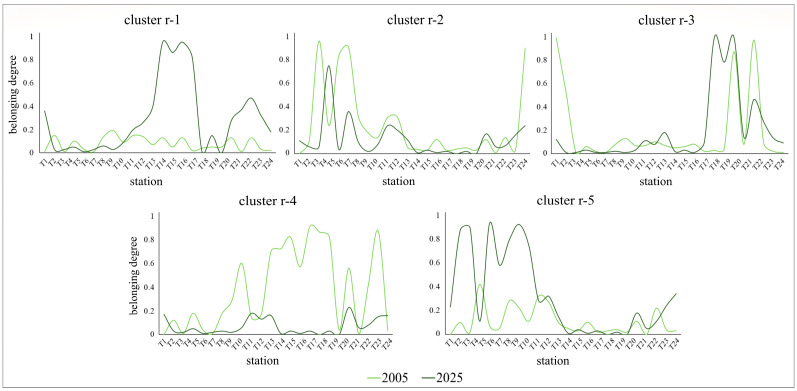
Belonging degree of each river station to the five species groups identified (fuzzy clusters) in the riparian sector with respect to the 2005 (light green line) and 2025 (green line) surveys. Dominant species in the groups are: *Phalaris arundinacea*, *Acer negundo*, *Amorpha fruticosa*, *Populus x canadensis* (r-1), *Phragmites australis*, *Populus alba* (r-2), *Phalaris arundinacea* (r-3), *Lythrum salicaria*, *Echinochloa crus-galli* (r-4), *Amorpha fruticosa*, *Populus alba* (r-5). For graphical representation, a smoothed line graph was used instead of a bar graph, for the sole purpose of facilitating data visualization.

By analyzing the distribution of each identified species group, it was possible to reconstruct the changes over time in these species assemblages along the investigated river stretch (Figure 5 and Figure 6). In the aquatic sector (Table 1), group a-1, dominated by *Myriophyllum spicatum,* and secondarily by *Stuckenia pectinata*, shows a wide and continuous distribution in 2005, especially from the urban stretch (from T12 to T23), whereas in 2025 it assumes a more fragmented distribution, restricted to only some stations (Figure 5). Group a-2, characterized by *Ceratophyllum demersum*, is poorly represented in 2005, while it shows significant peaks at some stations (e.g., T9, T11, T20) in 2025 (Figure 5), where it appears to produce new and extended populations. Group a-3, mainly characterized by *S. pectinata*, and secondarily by *M. spicatum*, shows in 2005 a discontinuous distribution with well-defined peaks along the entire river stretch, whereas in 2025 it is mainly represented starting from the urban stretch within Rome, where it shows high values distributed over wider intervals (Figure 5). Group a-4, mainly dominated by *Potamogeton nodosus*, shows in both surveys a distribution concentrated mainly in the pre-urban stretch; however, in the most recent survey, it displays higher and broader peaks (Figure 5). Except for some species groups, a moderate temporal stability of species assemblages in the aquatic sector generally emerges, a result also confirmed by the Boolean partition of stations (Table S2), which highlights that almost 50% of the sampled stations maintain the same species assemblage over time.

In the riparian sector, five main groups of species were identified (Table 1). Group r-1, dominated by the alien species *Amorpha fruticosa*, *Acer negundo*, *Populus x canadensis* and by the native *Phalaris arundinacea*, is almost absent along the investigated river stretch in the 2005 survey, whereas in 2025 it is widely distributed, especially along the urban and downstream stretch (Figure 6). Group r-2, characterized by *Phragmites australis* and *Populus alba*, shows in both surveys a spatial pattern mainly concentrated in the uppermost river stretch, but with a tendency to contract over time (Figure 6). Group r-3, mainly driven by *Phalaris arundinacea*, exhibits a substantially similar distribution between the two surveys (Figure 6), indicating temporal stability along the investigated river stretch. Group r-4, dominated by *Lythrum salicaria* and *Echinochloa crus-galli*, is widely distributed along the river gradient in 2005, with high peaks at numerous stations, while in 2025 it shows values close to zero at all sampled stations (Figure 6). Group r-5, characterized by the alien species *Amorpha fruticosa* and the native *Populus alba*, increases significantly in distribution over time along the pre-urban stretch (i.e., T1–T9) (Figure 6). The significant temporal variations observed in riparian species groups (r-1 and r-5 expanding, r-2 and r-4 contracting) indicate an overall instability of this river sector, a pattern also confirmed by the Boolean partition (Table S2), according to which the riparian floristic assemblage retains the same species group over time at only three stations (i.e., T10, T19, and T21).

### 2.4. Temporal Floristic Changes at the Single Species Level

**Figure 7:**
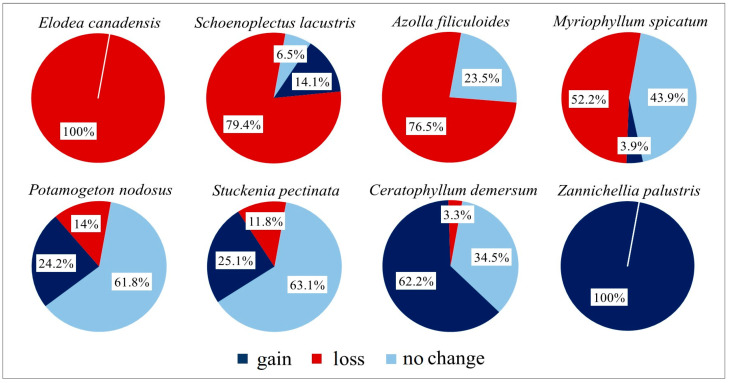
Temporal changes in the total coverage of the most frequent aquatic species recorded along the investigated river stretch. Gain, loss and no change represent the percentages of coverage gained, lost, and maintained by each species, respectively.

Comparison between the two aquatic floristic surveys (2005, 2025) based on the coverage values of the found hydrophytes, highlighted a series of temporal changes in terms of abundance and frequency of these species (Figure 7). The plant coverage of some hydrophytes, such as *Zannichellia palustris* and *Ceratophyllum demersum*, have increased over time, with a prevalence of gain over loss in the latest survey, whereas the coverage of *Myriophyllum spicatum*, *Schoenoplectus lacustris* and the aliens *Elodea canadensis* and *Azolla filiculoides* has decreased. *Stuckenia pectinata* and *Potamogeton nodosus* have maintained consistently high coverage values over time.

Nineteen aquatic species were exclusive to the most recent survey (i.e., 2025: gain 100%), as they had not been previously recorded locally; among these, five are invasive alien species (*Alternanthera philoxeroides*, *Amorpha fruticosa*, *Bidens frondosa*, *Lemna minuta*, *Paspalum distichum*), which nevertheless show limited frequency (≤3 sites out of 24). Species exclusive to the 2005 survey, as they were not recorded in the most recent one (i.e., 2025: lost 100%), are *Persicaria amphibia*, *Potamogeton trichoides*, and the alien *Elodea canadensis*, the latter already showing low mean coverage values (3.0%) in the previous survey.

**Figure 8:**
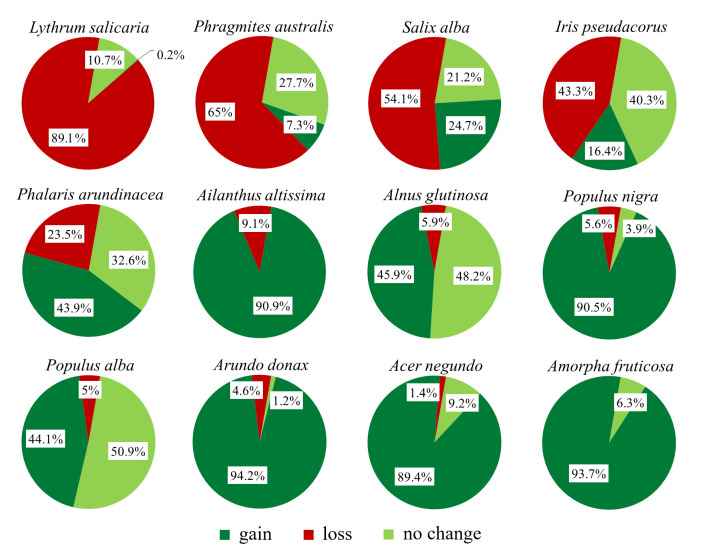
Temporal changes in the total coverage of the most frequent riparian species recorded along the investigated river stretch. Gain, loss and no change represent the percentages of coverage gained, lost, and maiby each species, respectively.

With reference to the riparian sector, among the 88 species in common between the two surveys (2005, 2025), 36 species increased over time both in terms of mean coverage and frequency (i.e., 2025: gain > loss) (e.g., *Phalaris arundinacea*, *Populus alba*, *Populus nigra*, *Alnus glutinosa*, *Amorpha fruticosa*, *Erigeron sumatrensis*, *Acer negundo*, *Arundo donax*, *Ailanthus altissima*), 31 species decreased (i.e., 2025: gain < loss) (e.g., *Bidens frondosa*, *Lythrum salicaria*, *Lycopus europaeus*, *Phragmites australis*, *Salix alba*, *Iris pseudacorus*) and 21 species remained almost stable (no change) (Figure 8). Among the gain species, *Amorpha fruticosa*, besides becoming the most frequent invasive alien species along the investigated river stretch, showed a marked increase in mean coverage over time, rising from 1.1% to 13.2% in the occupied stations. A group of 92 riparian species was found to be exclusive to the most recent survey (i.e., 2025: gain 100%), of which 16% are alien species, and almost 80% are represented by species weakly linked to the riparian environment, among which the ruderal *Symphyotrichum squamatum* (13/24 stations) and *Jacobaea erratica* (12/24 stations). Conversely, 27 species were found exclusively in 2005 (i.e., 2025: loss 100%), of which 86% were typical riparian species, including *Lythrum salicaria* (reducing from 21 stations with mean coverage of 12.2% to 11 stations and 1.4%), *Bidens frondosa* (from 22 stations with 4.0% coverage to 15 stations and 1.1%) and *Lycopus europaeus* (from 20 stations with coverage of 3.2% to 13 stations and 1%) (Figure 8).

### 2.5. Multitemporal Floristic Analysis of Ecological Traits

**Figure 9:**
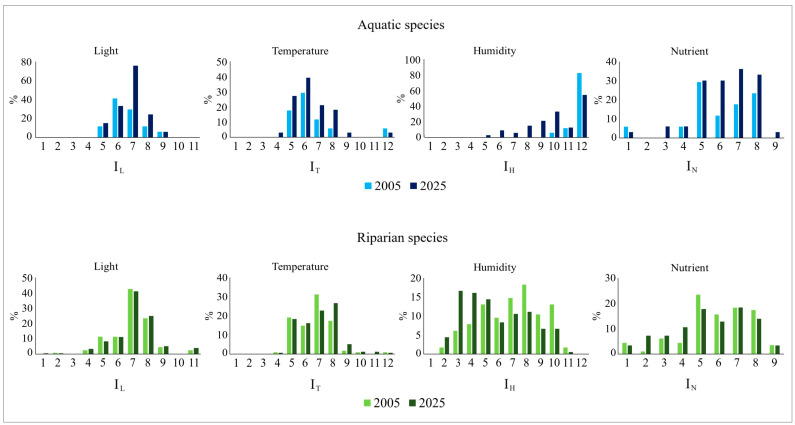
Percentage values (%) of Ellenberg’s indices related to light (I_L_), temperature (I_T_), humidity (I_H_), and nitrogen content (I_N_), calculated for the aquatic and riparian plant communities in the two surveys (2005, 2025).

Comparing the two floristic surveys (2005, 2025) from an ecological viewpoint, there have been changes in the ecological spectrum of the plant communities over time (Figure 9). Specifically, in the aquatic sector, there has been a significant increase in heliophilous (I_L_ > 7), thermophilous (I_T_ > 7) and nitrophilous (I_N_ > 6) species. Conversely, strictly hydrophilous species, i.e., constantly submerged in water (I_H_ = 12), such as *Potamogeton nodosus*, *Myriophyllum spicatum* and *Ceratophyllum demersum*, decreased from 82.4% to 54.5%, while species associated with intermediate to moderately high humidity values (5 ≤ I_H_ ≤ 10), such as *Arundo donax*, *Agrostis stolonifera*, *Galium palustre* and *Polypogon viridis*, increased (Figure 9).

With reference to the riparian sector, no significant temporal variations were detected with respect to the light factor or edaphic nitrogen availability, whereas increases over time were recorded for thermophilous (I_T_ > 8) and xerophilous species, i.e., species adapted to dry soils (I_H_ < 5), accompanied by a parallel decrease in the percentage of hygrophilous species (I_H_ > 6) (Figure 9). Restricting this type of analysis to two subsets of riparian lost species with respect to the last survey and recently arrived species (new species), no significant difference emerged between the two subsets with regard to the light factor. Instead, for the temperature factor, the subsets of lost and new species showed higher percentages at intermediate (I_T_ = 5–7) and higher (I_T_ > 8) index values, respectively. Among new species, xerophilous species prevail, i.e., those with low values of the humidity index (I_H_ = 3–4). With reference to nutrients, lost species are mostly associated with moderately rich environments (I_N_ > 5), whereas new species, while maintaining substantial occurrences at the same index values as lost species, display a broader value distribution that also includes lower ones (I_N_ = 2–4).

### 2.6. Biological, Chorological and Environmental Compatibility Analysis of the Riparian Plant Component

The comparative analysis of the biological spectra calculated for the plant component sampled at the two time points (2005, 2025) highlights an increase over time in therophytes (from 18.3% to 25%) and hemicryptophytes (from 33.9% to 40%), accompanied by a parallel decrease in geophytes (from 19.1% to 13.9%) and phanerophytes (from 21.7% to 17.2%). The morphotypes more closely linked to the river environment, such as helophytes and rooted hydrophytes, also decreased over time or locally disappeared. The calculation of the T/(H + G) ratio on the overall datasets showed a general increase in this index, rising from 0.34 in 2005 to 0.46 in 2025. The index reaches an even higher value (0.58) when calculated for the subset of new species found in the 2025 survey, in which therophytes account for 32%.

Comparing the chorological spectra calculated for the two plant communities sampled at time points (2005, 2025), it emerges that over the last twenty years the plant community recorded along the riverbanks has undergone a general increase in Mediterranean species (steno- and euri-Mediterraneans increased from 5.2% and 7% to 10% and 12.8%, respectively). An increase was also recorded for multizonal species, which rose from 20% to 26.7%. Although the percentage of alien species shows a little decrease (from 21.7% to 19.4%), their absolute number increased significantly over time (from 25 to 35 species). Species more closely linked to continental and cool climates, such as Eurasian and Boreal species, decreased over time, ranging from 33% to 21.7% and from 10.4% to 6.7%, respectively.

Regarding the ratio between typically riparian and generalist species, it decreased over time, from 2.1 in 2005 to 0.7 in 2025. A similar, but even more pronounced, trend was obtained considering the subset of new species recorded in 2025, for which this ratio is even lower (0.2), with only 16% of the new species characteristic of riverbanks. Conversely, the subset of species no longer locally found in the latest floristic surveys shows a relatively higher indicator value (2.38), with 70% of the lost species closely associated with the riparian environment.

## 3. Discussion

A comparison between two floristic surveys carried out in permanent plots 20 years apart (2005, 2025) highlighted important temporal floristic changes in terms of α-diversity and temporal β-diversity, as well as in single species and species assemblage frequency, abundance, composition and distribution along the lower stretch of the Tiber River. These floristic changes were analyzed using an integrated approach, analyzing the species dominance-abundance patterns together with distributive, chorological, biological, and ecological aspects of both the aquatic and riparian plant communities.

### 3.1. Independence Between the Aquatic and Riparian Plant Components

Comparative floristic analyses highlighted that the aquatic and riparian plant communities respond independently to the environmental variables of the investigated river stretch. In particular, in the aquatic sector, temporal β-diversity generally decreased along the river stretch (loss > gain), indicating that losses, both in terms of species disappearance and decreases in plant coverage, exceeded gains over time (Figure 3), despite the increase in α-diversity recorded between 2005 and 2025 (Figure 1). Conversely, in the riparian sector, temporal β-diversity generally increased over time (loss < gain) (Figure 3). Indeed, during the time period considered, there was both a greater arrival of new species compared to those lost locally, with an effective increase in α-diversity as well, and an increase in plant coverage along the riverbanks. It should be noted, however, that this increase mainly involved species, such as *Phalaris arundinacea*, *Plantago major* and *Dittrichia viscosa*, i.e., cosmopolitan, weedy, ruderal or generalist species weakly linked to the riparian environment. Furthermore, these species have often been found associated with invasive alien species, such as *Amorpha fruticosa* and *Acer negundo*, known for their high rates of reproduction and seed dispersal [23,24,25,26,27].

These various responses shown by aquatic and riparian plant communities are likely due to the different environmental pressures to which these two different plant communities are subjected. Indeed, in aquatic environments, plants respond to specific pressures, such as hydrological alterations (e.g., variations in discharge and flow velocity) and changes in water chemical–physical characteristics (e.g., eutrophication, thermal increase), which often lead to a reorganization of aquatic flora, with replacement and/or rarefaction and disappearance of the most sensitive taxa [28]. Along the riverbanks of Mediterranean and disturbed watercourses, such as the investigated stretch of the Tiber River, the phenomenon of riparian vegetation encroachment (RVE) [29,30] is often observed, according to which reductions in flood frequency and river discharge make new surfaces available along riverbanks for colonization by additional plant species. Moreover, anthropic disturbance and morpho-structural alteration of riverbanks (e.g., dams, containment works, channelization, mowing) promote over time the fragmentation of existing plant communitiesand the formation of new microhabitats that allow the arrival and spread of new species along the riverbanks. The combined effect of these pressures is most likely responsible for the observed increase in α-diversity, temporal β-diversity, and total plant coverage along the riverbanks of the investigated river stretch.

### 3.2. Temporal Changes in Plant Species Assemblages

Identifying different species assemblages with distinct temporal distribution patterns along the investigated river stretch made it possible to reconstruct local changes in the aquatic and riparian plant communities, providing evidence of main environmental changes occurring along the river over the last two decades (2005–2025). As regards the aquatic community, the most frequent species in both floristic surveys were *Stuckenia pectinata* and *Myriophyllum spicatum* (groups a-1 and a-3), i.e., bioindicator species of eutrophic, polluted and low quality waters [10,31,32]. In the most recent survey, however, the higher frequency and coverage of these species, as well as of the euriecious *Ceratophyllum demersum* (group a-2), especially in the urban and terminal stretches of the river, indicate that issues of water eutrophication and pollution remained largely unresolved over time in this stretch of the river. A different situation appears to have occurred in the pre-urban river stretch, where the wider distribution than in the past of populations dominated by *Potamogeton nodosus* (group a-4), that is a species less markedly eutrophic than those mentioned above, would suggest a relative attenuation of water eutrophication in that river stretch.

Along the riverbanks, especially in the urban and terminal stretches, the recent wide distribution of populations dominated by the native *Phalaris arundinacea* (groups r-1 and r-3), and the alien species *Amorpha fruticosa* and *Acer negundo* (group r-1), suggests that over time there has generally been an increase in anthropic disturbance and biological pollution in this river sector. Indeed, these species are known for their weedy, ruderal, and in the case of the two alien species, invasive nature, being able to rapidly colonize unstable and disturbed areas and to strongly compete with native species [23,33,34]. In particular, *A. fruticosa* appears to successfully compete with native riparian species due to its ability to modify edaphic conditions to its advantage and to produce and release into the soil allelopathic substances that inhibit the growth and seed germination of the other plants [27,34,35]. Conversely, populations characterized by typical riparian native species, such as *Phragmites australis* and *Populus alba* (group r-2), and *Lythrum salicaria* and *Echinochloa crus-galli* (group r-4), showed a marked contraction over time, probably due to fragmentation and loss of suitable riparian habitats caused by anthropic pressures [36], as well as to invasion by competitive alien species, such as the above-mentioned *A. fruticosa* and *A. negundo*.

Overall, the persistence of the species assemblages identified along the riverbanks is very low, as out of 24 stations only three maintain the same type of species assemblage in both floristic surveys (2005, 2025), confirming the high dynamism of the riparian plant communities, which reflects the equally high environmental instability associated with widespread anthropic disturbance along the banks in this river stretch. Conversely, in the aquatic sector, a greater general stability is observed, although the tendency toward contraction (group a-1) and expansion of some species assemblages (groups a-3, a-4) (Figure 5) nevertheless highlight a redistribution and reorganization of aquatic plant communities over time.

### 3.3. Temporal Changes in River Plant α-Diversity

The comparison between the two floristic surveys (2005, 2025) revealed a substantial increase in floristic richness over time, both in the aquatic (+94.1%) and riparian (+56.5%) sectors. However, this increase is mainly due to the arrival of new species that in most cases are not typical of the river environment. This is particularly evident along the riparian sector, where the arrival of numerous ruderal, multizonal and generalist species (e.g., *Digitaria sanguinalis*, *Dittrichia viscosa*, *Urtica dioica*), as well as alien species (e.g., *Amorpha fruticosa*, *Dysphania ambrosioides*, *Erigeron sumatrensis*), was recorded. The widespread diffusion of these species has occurred at the expense of some native species typically associated with riparian environments (e.g., *Alisma plantago-aquatica*, *Butomus umbellatus*, *Scutellaria galericulata*, *Typha angustifolia*, *Veronica beccabunga*, *Phragmites australis*, *Salix alba*).

These results indicate a condition of erosion of the typical riverine flora, presumably due to the intensification over time of various anthropogenic pressures that impact the investigated river stretch, including tourism and recreational river use, sporting activities, mowing and chemical control of weeds, bank alteration and introduction of alien species [30,37]. It is known, in fact, that anthropogenic disturbance causes environmental instability, reducing the resistance of the ecosystem and favoring the spread of generalist, tolerant and alien taxa, which often successfully outcompete specialized and native plants [38,39,40,41]. Furthermore, in this study, the increase in the absolute number of alien species in the last twenty years, both in the aquatic (from 2 to 6 species) and riparian sectors (from 25 to 35 species), confirms previous investigations that highlighted the vulnerability of the river ecosystems to biological invasions [42,43,44].

### 3.4. Temporal Changes in Ecological Traits of Aquatic and Riparian Plants

Significant ecological differences also emerged between the two floristic surveys (2005, 2025). In particular, in the aquatic sector, a general increase over time was recorded in nitrophilous, heliophilous, and thermophilous species. Similarly, in the riparian sector, the frequency/abundance of heliophilous and thermophilous species increased, as well as that of xerophilous species, i.e., species adapted to dry soils.

The increase in nitrophilous, heliophilous, thermophilous and xerophilous species could suggest that, in the last twenty years, some processes have intensified along the river stretch considered, and in particular: (i) increase in organic and nitrogen content in water, i.e., in the level of water eutrophication, (ii) shading reduction of the river environment, consequent to the intensification of mowing activities and the partial local removal of the tree-shrub riparian vegetation, (iii) increase in air and water temperature, as a consequence of the aforementioned reduction in the shading conditions of the riverbed, global warming linked to climate change, and the urban heat island effect, particularly relevant in the river stretch crossing the urban area [45], (iv) reduction in the rainfall regime and floods, due to ongoing climate change [46], with consequent drying out of the soils along the riverbanks. These changes, mostly of anthropogenic origin, may have modified the environmental characteristics of both the aquatic and riparian sectors, resulting in more eutrophic waters and warmer, xeric, and sunnier local conditions. Indeed, the most recent floristic survey recorded a greater spread of eutrophic and Mediterranean species, i.e., species associated with hot, arid conditions; conversely, a reduction was observed in typically riparian and Eurasian species, which are species associated mainly with cool, humid environmental conditions. A comparison between the group of locally extinct species and new ones found along the riverbanks confirms this trend; in fact, the new species are more thermophilic and xerophilic than the extinct ones.

### 3.5. Methodological Considerations and Future Research Directions

A multitemporal floristic comparison of permanent plots, based on α- and temporal β-diversity analyses, provides valuable long-term insights for assessing changes in plant communities over time. However, this well-established and widely adopted approach [47,48] might not fully capture the spatial heterogeneity of plant communities at the local scale [49] or the rapid changes that specifically characterize aquatic and riparian environments [50].

To overcome these limitations, future studies could combine the multitemporal approach adopted here with remote sensing techniques [51,52] or trait-based modeling approaches [53,54], which could help capture plant patterns at larger spatial scales and improve the interpretation of plant responses to environmental changes. An integrated approach combining these tools with traditional floristic analyses would provide both a more comprehensive assessment of freshwater ecosystem dynamics and enable more accurate detection of ongoing environmental changes.

## 4. Materials and Methods

### 4.1. Study Area

The Tiber River, with a length of approximately 400 km, represents the main river in peninsular Italy. It originates in the Tuscan-Emilian Apennines and flows mainly through the Umbria (upper–middle stretch) and Lazio region (lower stretch) before emptying into the Tyrrhenian Sea. From a biogeographical viewpoint, the Tiber River basin falls within the Mediterranean district on the middle Tyrrhenian side [55].

**Figure 10:**
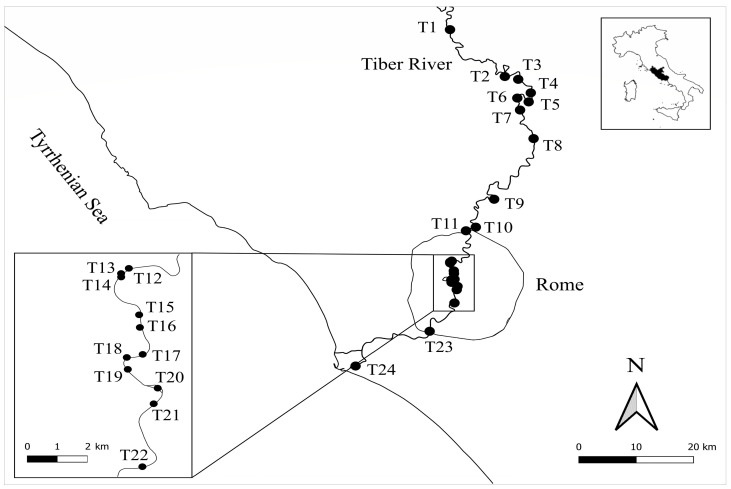
Study area. Location of the 24 sampling river stations selected along the lower stretch of the Tiber River flowing through the Lazio region (central Italy). At the top right, within the border of Italy, the Lazio region is shown as a dark area, while at the bottom left there is a detail of the selected stations along the urban river stretch within the city of Rome.

The study focuses on the lower stretch of the Tiber River, extending from the area of Magliano Sabina to the river mouth near the urban center of Ostia (Figure 10). In this stretch, the Tiber initially flows through a territory characterized by predominantly agricultural land use, and then crosses the “Tevere-Farfa Regional Natural Reserve” area, where the riverbed widens due to the presence of a downstream hydroelectric dam. Downstream from the Reserve and up to the outskirts of the city of Rome, there is a progressive increase in residential settlements and industrial areas. Within Rome, in the central urban sector, the river flows between artificial banks and, here, tourist and sporting activities in the water and along the banks, as well as periodic mechanical mowing or soil alteration along the banks, cause instability and environmental alteration; in the peri-urban areas, and then towards the mouth, the presence of semi-natural banks offers more space for vegetation growth, which is nevertheless limited by some anthropogenic pressures, such as agricultural, fishing, shipyard activities, and residential settlements along the riverbank [14,18].

### 4.2. Plant Sampling Methodology

Plant sampling was performed in 2025 from May to September, corresponding to the main seasonal growth period of aquatic and riparian plants. Qualitative and quantitative floristic surveys were carried out at 24 river stations selected along the Tiber River, coinciding with those sampled (permanent plots) in a similar study performed previously in 2005 [18], using the same sampling methodology. The georeferencing of the stations was conducted using QGIS software vers. 3.34.2 (QGIS Development Team, Open Source Geospatial Foundation, Chicago, IL, USA) (Table S3 and Figure 10).

Floristic surveys were conducted separately in the aquatic and riparian sectors, within plots (10 m × 3 m) arranged parallel to the river stretch, for a total of 48 surveys. In each plot, both total plant coverage (%) and that of each recorded plant species were visually estimated. All spontaneous native and alien species were sampled, whereas cultivated or planted species were not included in the sampling. For the most critical plant taxa, some specimens were collected in the field and subsequently transported to the laboratory for taxonomic identification under a stereoscope (Zeiss Stemi 305, White Plains, NY, USA), using dichotomous keys for the Italian vascular flora [56].

### 4.3. Data Analysis

The floristic data, collected during the two different time points (2005, 2025) at the permanent plots selected, were compared in terms of α- and temporal β-diversity, species composition, biological structure, chorology, ecology and species assemblages, in order to highlight any floristic changes that have occurred over time along the investigated river stretch.

### 4.3.1. Species Assemblage Analyses

Comparing the floristic surveys carried out in 2005 (05) and 2025 (25), all plant data collected in the permanent plots of the 24 selected stations were organized into two matrices, keeping the aquatic (A) and riparian (R) communities separate. The resulting data matrices analyzed were: A (36 aquatic species × 48 aquatic plots) and R (207 riparian species × 48 riparian plots).

The quantitative analyses to identify changes over time in plant species assemblages in the permanent plots followed two different approaches. The first was based on the analysis of temporal β-diversity, following the definitions of Legendre [16] and Magurran et al. [15], decomposing the observed changes into distinct indicators of temporal β-diversity (TBI): gain (TBI gain), i.e., arrival of new species or increases in plant abundance, loss (TBI loss), i.e., local extinction or decreases in plant abundance, total (TBI total) and persistence of a common part (no change) [57,58]. To quantify temporal β-diversity, the Bray-Curtis index was used as suggested by Legendre [16], which measures the percentage dissimilarity between species assemblages. The common part was obtained by calculating the reciprocal of 100 of the total dissimilarity. The gain and loss indicator values calculated for each plot in both aquatic and riparian systems were analyzed through Principal Component Analysis (PCA), using correlation coefficients and biplot method [59]. The statistics of gain and loss of every single species, always obtained from the Bray-Curtis index, were calculated to measure changes in the population of such species.

The second approach was based on clustering methods [60] to analyze the data structure and identify floristically homogeneous groups of permanent plots over time. Specifically, fuzzy clustering was applied separately to matrices A and R in order to obtain the optimal partition of the datasets into homogeneous groups of aquatic and riparian systems. The algorithm was based on fuzzy set theory [61], where each group is represented as a fuzzy cluster and the membership of each object is expressed as a continuous value between 0 and 1 (belonging degree) [62,63]. The classification of the stations was performed by using fuzzy c-means clustering [62,64] on the chord distance matrix. The optimal partition, showing the best number of fuzzy clusters according to the floristic data assemblage, was determined through the normalized Dunn coefficient [65]. The centroid values of the single cluster were calculated by a weighted average (weights were the belonging degree to such cluster of the plots) of every species. Also, a Boolean partition was obtained by assigning each station to the cluster with the highest belonging degree [66]. Finally, the temporal behavior of the clusters was visualized by plotting the membership functions of stations along the river stretch for the two time points (05, 25). The comparison of these two series highlighted the changes in the belonging degree and, consequently, the dynamics of the floristically homogeneous groups.

Statistical and graphic outcomes of such analyses were obtained using the Ginkgo package vers. 1 (University of Barcelona, Spain) [64] and Microsoft Excel vers. 2409 (Microsoft Corporation, Redmond, WA, USA), respectively.

### 4.3.2. Species Traits Analyses

For each plant species recorded in aquatic and riparian sectors, native or alien status in Italy was assigned according to the Acta Plantarum website [67] to calculate the percentage of native and alien species (%) and their numerical ratio (alien/native) in each station in the two different surveys (2005, 2025). This is useful for assessing whether biological pollution has increased or decreased over time in the river stretch investigated.

For each species, values of Ellenberg autoecological indices [68], adapted to the Italian flora [12,69], and referring to light (I_L_), temperature (I_T_), humidity (I_H_), and nutrients (I_N_), were assigned for calculating the ecological spectra of the aquatic and riparian plant communities found in the two surveys.

For the riparian plant community, chorological and biological spectra were also calculated. To do this, each riparian species was assigned its chorotype and morphotype according to Pignatti [56]. A floristic index based on the ratio between therophytes (T) and hemicryptophytes and geophytes (T/H + G) was calculated; this index represents a variant of the T/H index used in literature [70,71] and is considered a good indicator of the degree of anthropization of an area according to a direct proportionality between increasing index values and anthropic disturbance.

In addition, the ecological compatibility with the riparian environment of each species found along the riverbanks was also evaluated, considering both the authors’ expertise and the value of the Ellenberg humidity index (I_H_). According to I_H_, higher values, on a scale from 1 to 12, are assigned to species strictly linked to wet environments, which in this context indicates a higher compatibility with the riparian environment. The numerical ratio between typical riparian and generalist species was used as a floristic indicator of environmental compatibility, and therefore of naturalness. Biological, chorological and environmental compatibility analyses were not conducted on the aquatic plant community as this is considered particularly uniform both biologically, being characterized exclusively by hydrophytes, and chorologically, being notoriously dominated by multizonal, azonal species, i.e., species weakly linked to the climatic conditions of a territory and more closely related to the hydrological characteristics of the waterbody [72,73]. Similarly, from the environmental compatibility viewpoint, the species found in the aquatic sector are necessarily linked to the aquatic environment, making the application of the floristic environmental compatibility indicator poorly meaningful.

All these analyses and graphic outcomes were obtained by using the Microsoft Excel vers. 2409.

## 5. Conclusions

The multitemporal study carried out along the lower stretch of the Tiber River highlighted significant local floristic changes over the last twenty years both in terms of α- and temporal β-diversity as well as in plant species ecological, chorological, biological traits and species composition of the plant assemblages recorded in aquatic and riparian sectors. However, it is noteworthy that these two sectors have shown different patterns of floristic change. Overall, the aquatic plant community has proven more stable over time than the riparian ones, which has instead shown greater changes under the multiple environmental pressures affecting riverbanks. In general, the most recent survey recorded a richer flora but characterized by the presence of generalist, ruderal and alien species. These have partially replaced, especially in the riverbank area, species typical of the river environment, leading to processes of simplification and homogenization of the plant communities of the studied river stretch.

A floristic comparison between the two surveys from a chorological, ecological and biological viewpoint highlighted an increase in human disturbance along the river over time. The loss of some native, Eurasian, hygrophilous, and perennial species, on the one hand, and the spread of multizonal, alien, xerophilous and short-lived species, on the other, indicate an intensification of conditions of alteration and significant environmental instability along the investigated river stretch, particularly in the riparian sector.

Overall, the results of this study highlighted the need to implement management actions that must be aimed, on the one hand, at restoring the most degraded areas of the river stretch investigated and, on the other, considering the floristic peculiarities and criticisms of riparian and aquatic environments. Specifically, in the riparian sector, the replacement of typical riparian native species with ruderal and invasive alien ones highlights the need to reduce anthropic disturbance along the banks (e.g., avoiding mechanical plant removal, bank structural alteration, agriculture and industrial activities close to the banks), and to check and monitor carefully alien species [74], particularly those showing a highly invasive character, such as the American *Amorpha fruticosa* and *Acer negundo*. In the aquatic sector, the spread of eutrophic and pollution-tolerant species, along with the decline of more sensitive taxa, highlights the importance of reducing local pollution sources by better regulating discharges and improving water quality along the river stretch under consideration. Overall, management actions aimed at restoring more natural hydromorphological and environmental conditions would help reclaim the most degraded areas and recover the ongoing process of banalization and erosion of native aquatic and riparian flora, in line with recent environmental recovery assessments along the urban stretch of the Tiber River [75].

In conclusion, the multitemporal floristic analysis approach adopted in this study proved to be a useful tool for identifying, monitoring and interpreting the main environmental changes that occurred along the river over time. This provides important reference knowledge to support conservation and environmental restoration policies for river stretches of high historical, economic, naturalistic, and landscape value, such as the stretch of the Tiber River investigated here.

Possible future research in which the methodological approach used here is integrated with complementary analytical tools, such as remote sensing or plant trait-based modeling, could improve the accuracy of the assessments and provide a more comprehensive understanding of the environmental dynamics of river ecosystems over time.

## Data Availability

The original contributions presented in the study are included in the article/Supplementary Materials.

## Supplementary Materials

The following supporting information can be downloaded at https://www.mdpi.com/article/10.3390/plants15050716/s1, Table S1a: Temporal β-diversity (TBI) indicators (total, loss, gain, no change) for the sampled stations; Table S1b: Mean values of temporal β-diversity indicators (total, loss, gain, no change) calculated for the aquatic (A) and riparian (R) sectors. Statistically significant values (*p* < 0.01) are shown in bold; Table S2: Boolean partitions of stations derived from aquatic and riparian fuzzy partitions. Numbers in the cells represent the cluster with the maximum belonging degree according to the corresponding fuzzy partition. Stations that do not change their memberships between the two surveys considered (2005, 2025) are highlighted in gray; Table S3: List of the sampling stations along the lower stretch of the Tiber River. For each station, code, locality and geographic coordinates (latitude, longitude) are reported. Stations are arranged along an upstream-downstream gradient, following the numerical order from 1 to 24.
